# Serum miR-375 for Diagnostic and Prognostic Purposes in Medullary Thyroid Carcinoma

**DOI:** 10.3389/fendo.2021.647369

**Published:** 2021-03-29

**Authors:** Simona Censi, Loris Bertazza, Ilaria Piva, Jacopo Manso, Clara Benna, Maurizio Iacobone, Alberto Mondin, Mario Plebani, Diego Faggian, Francesca Galuppini, Gianmaria Pennelli, Susi Barollo, Caterina Mian

**Affiliations:** ^1^ Endocrinology Unit, Department of Medicine (DIMED), University of Padua, Padua, Italy; ^2^ Endocrine Surgery Unit, Department of Surgical, Oncological and Gastroenterological Sciences (DiSCOG), University of Padua, Padua, Italy; ^3^ Laboratory Medicine, Department of Medicine (DIMED), University of Padua, Padua, Italy; ^4^ Surgical Pathology and Cytopathology Unit, Department of Medicine (DIMED), University of Padua, Padua, Italy

**Keywords:** miR-375, circulating miRNAs, medullary thyroid cancer, calcitonin, diagnostic marker

## Abstract

**Purpose:**

Having previously demonstrated that tissue miR-375 expression in medullary thyroid carcinoma (MTC) tissues is linked to prognosis, the aim of this study was to assess the diagnostic and prognostic value of circulating miR-375 levels in MTC patients.

**Methods:**

A series of 68 patients with MTC was retrospectively retrieved and assessed in terms of their clinicopathological characteristics. MiR-375 levels were measured in all patients’ presurgical blood samples. Both serum and tissue levels were tested prior to surgery in a subgroup of 57 patients. Serum miR-375 levels were also measured in serum from 49 patients with non-C-cell thyroid nodular diseases (non-CTN), 14 patients with pheochromocytoma, and 19 healthy controls.

**Results:**

Circulating miR-375 levels were 101 times higher in the serum of patients with MTC than in all other patients and controls, with no overlap (P < 0.01). No correlation emerged between serum and tissue miR-375 levels. Serum miR-375 levels were higher in MTC patients with N0 than in those with N1 disease (P = 0.01), and also in patients who were biochemically cured than in those who were not (P = 0.02). In the whole series of patients and controls, calcitonin (CT) and serum miR-375 levels were correlated at diagnosis (R^2^ = 0.40, P < 0.01), but in a U-shaped manner: a positive correlation was found with low CT levels, then the correlation turns negative as CT rises (in MTC patients). A negative correlation was indeed found in MTC patients between serum miR-375 and CT (R^2^ = −0.10, P = 0.01). On ROC curve analysis, a cut-off of 2.1 for serum miR-375 proved capable of distinguishing between MTC patients and the other patients and controls with a 92.6% sensitivity and a 97.6% specificity (AUC: 0.978, P < 0.01).

**Conclusions:**

Serum miR-375 levels can serve as a marker in the diagnosis of MTC, with a remarkable specificity. Serum miR-375 also proved a novel marker of prognosis in this disease. Further *in vitro* experiments to corroborate our results are currently underway.

## Introduction

Medullary thyroid cancer (MTC) is a neuroendocrine neoplasm arising from thyroid parafollicular C-cells.

Sporadic MTC (sMTC) carries somatic REarranged during Transfection *(RET)* mutations in approximately 50% of cases, with a subset of sporadic and *RET*-negative MTC also carrying a mutation in *RAS* genes (1). It is well known that somatic *RET* mutations point to a poor prognosis in sMTC, and somatic *RAS* mutations to a better prognosis (2). It is therefore useful to assess patients’ mutational status for prognostic purposes, and crucial to the choice of new target treatments such as the now-approved tyrosine kinase cabozantinib (3, 4) or the highly selective *RET* inhibitor pralsetinib currently being trialed for use against MTC (5). About 40–60% of sporadic MTCs do not carry any recognized genetic driver, however, making it difficult to establish a patient’s prognosis and therapeutic options. In short, the discovery of new molecular changes remains pivotal to improving the prognostic stratification of patients with MTC, and to the search for novel targets for therapy.

The availability of new serum markers would also benefit the diagnosis of MTC. Calcitonin (CT) is a 32-amino-acid monomeric peptide produced by C-cells. It is a sensitive marker for the purposes of tumor diagnostics and prognostics because its serum concentrations correlate directly with the C-cell mass (6). Measuring CT has many pre-analytical, analytical, and post-analytical pitfalls, however, which sometimes make its interpretation difficult, especially in the event of moderately elevated levels (7).

One in four cases of MTC—hereditary MTC, hMTC—arises in the context of an autosomal dominant multiple endocrine neoplasia syndrome type 2 (MEN2), which is caused by *RET* proto-oncogene germline mutations (in both MEN2A and MEN2B) (8). Affected individuals initially develop primary C-cell hyperplasia (CCH), which progresses to early invasive MTC, and eventually to grossly invasive macroscopic MTC. Apart from patients with the *RET* codon M918T mutation (who should undergo thyroidectomy in the first year of life), the age of onset and aggressiveness of MTC varies considerably, even among individuals from the same family (4). Hence the particular interest in finding new serum markers to improve the specificity of patients’ diagnosis and prognosis, in both sMTC and hMTC.

MiRNAs are endogenous single-stranded non-coding RNAs that selectively bond to the complementary 3’UTR mRNAs, influencing their cleavage and translation (9) and many studies have documented their involvement in the pathogenesis of cancer, including endocrine tumors (10, 11). MiRNAs can act as “onco-miRNAs” or “oncosuppressor miRNAs,” their final biological function being tissue- and context-dependent (12). MiRNAs have also been isolated in biofluids, such as blood serum and plasma, and they have consequently emerged as novel biomarkers for use in cancer diagnostics and prognostics (13, 14–17). They have shown a remarkable stability in clinical samples of plasma and serum (17), possibly overcoming the analytical problems associated with CT measurement.

One of the most promising miRNAs involved in the pathogenesis of MTC is miR-375. A first study by our group demonstrated its overexpression in MTC, with levels 10 times higher than in normal thyroid tissues (18). In a subsequent study focusing on the tissue expression of miR-375 in a larger series of sMTC and hMTC, we confirmed that miR-375 levels are higher in MTC than in normal thyroid tissue, with no overlap in the levels measured between the two entities. We also documented a link between miR-375 tissue expression and the aggressiveness of a tumor’s clinicopathological characteristics and patient outcomes at the end of the follow-up. This would suggest a role for miR-375 as an onco-miRNA in the pathogenesis of MTC. Based on these findings in tissue miR-375, the aim of the present study was to examine circulating miR-375 levels, and their possible role in MTC diagnostics and prognostics.

## Materials And Methods

### Patients

A tissue bank has been operating at Padua University Hospital since 2005. Patients undergoing surgery for certain diseases (including nodular thyroid diseases and adrenal diseases) are routinely asked beforehand for permission to collect and store their tissue and serum samples for research purposes (protocol ref. 3388). All patients involved in this study thus gave their informed written consent to the banking of their tissue and serum samples, with the approval of the ethical committee for clinical experimentation at Padua Hospital. This study was conducted in accordance with the Declaration of Helsinki.

The study involved a consecutive series of 69 patients with MTC who underwent surgery between 2007 and 2020 (30 males and 39 females; median age: 55 years; range: 5–87 years). MiR-375 levels were assessed in all patients’ serum samples obtained at the time of surgery, before intervention. Analyses were conducted to identify all germinal and somatic *RET* mutations. Both preoperative serum and postoperative MTC tissue samples were available for a subgroup of 57 patients. For this subgroup, tissue miR-375 levels were also measured, and somatic *RET* and *RAS* mutations were sought.

Data were collected on CT levels at diagnosis, TNM staging at diagnosis, and the biochemical cure rate. The median follow-up was 70.5 months (IQR: 29.0–109.0 months).

For comparison, serum miR-375 levels were measured in samples from 49 patients with non-C-cell thyroid nodular diseases (non-CTN) (23 males and 26 females), whose histological diagnoses included: 12 follicular adenomas (FA); 15 hyperplastic nodules (HN); 10 follicular thyroid carcinomas (FTC); and 12 papillary thyroid cancers (PTC). MiR-375 was also assayed in serum samples obtained before adrenal surgery from 14 patients with pheochromocytoma (7 males and 7 females), and in the serum of 19 healthy controls (10 males, 9 females).

### RET Germline Mutations and RET/RAS Somatic Mutations

DNA was extracted from all patients’ serum samples and tissues frozen after surgery using the DNeasy Blood and Tissue kit (Qiagen, Milano, Italy), according to the manufacturer’s protocol. Analyses were performed by direct sequencing, as described elsewhere (19, 20): for *RET* (NM_020975.4; exons 5, 8, 10, 11, 13, 14, 15, and 16) in 47 tissue samples, and all serum samples; and for *N-RAS* (NM_002524.3; exons 2 and 3), *K-RAS* (NM_033360.2; exons 2 and 3), and *H-RAS* (NM_005343.2; exons 2 and 3) mutations in 42 tissue samples.

### miRNA Quantitative Real-Time Polymerase Chain Reaction

Total RNA was extracted from fresh snap-frozen samples of 57 MTC, using the TRIzol reagent as lysis buffer (Invitrogen, Carlsbad, CA, USA) according to the manufacturer’s protocol. RNA extractions from serum were performed using the Zymo DirectZol RNA Miniprep Plus Kit (cat. no. R2051) according to the manufacturer’s instructions. RNA was quantified by Nanodrop (Thermo-Fisher). CDNA synthesis was done with the TaqMan Advanced miRNA cDNA Synthesis Kit (Applied Biosystems, Milan, Italy).

A real-time quantitative PCR (qRT-PCR) was performed for has-miR-375-3p on the StepOne real-time PCR system using TaqMan advanced miRNA assays, and following the manufacturer’s instructions. Normalization was done through the application of the hsa-miR-24-3p. All real-time reactions, including no template controls, were run in triplicate. A pool of cDNA derived from mixed normal human thyroid tissues and serum was used as the calibrator source.

Data were analyzed with the relative quantification (2-ΔΔCt) method, as described elsewhere (21).

### Statistical Analysis

The Kolmogorov-Smirnov test showed that the variables were not distributed normally, so data are reported as medians and interquartile ranges (IQR). The Mann-Whitney test was used to analyze the tissue and circulating miR-375 serum levels and gender, lymph node involvement, stage of MTC at diagnosis (I+II *versus* III+IV), biochemical cure, *RAS* somatic mutation, *RET* somatic and germinal mutational status, and dichotomized CT levels. The Mann-Whitney test was also used to investigate the relationships: between CT levels, lymph node involvement, and stage of MTC at diagnosis (I+II *versus* III+IV); and between biochemical cure rate, CT levels, and tumor size. The Kruskal-Wallis test was used to evaluate miR-375 circulating levels with subjects’ condition (MTC *versus* non-CTN or pheochromocytoma or healthy controls). Categorical variables (biochemical cure rate, sex, age, and lymph node involvement) were compared with the chi-squared test. A P-value of <0.05 was considered statistically significant.

## Results

### Patients

**Table 1** shows the clinicopathological features of the MTC patients, including their mutational status and biochemical cure rates.

**Table 1:** Clinicopathological characteristics of the MTC patients enrolled.

	Parameter	Results
**Primary cancer size, median, IQR (mm)**		13, 8–24
**T**	1	35/63 (55.6%)
	2	11/63 (17.5%)
	3	16/63 (25.4%)
	4	1/63 (1.6%)
**Lymph node involvement**	N0	44/65 (67.7%)
	N1a	7/65 (10.8%)
	N1b	14/65 (21.5%)
**Tumor stage**	I	31/65 (47.7%)
	II	13/65 (20.0%)
	III	7/65 (10.8%)
	IV	14/65 (21.5%)
***RET* germline mutation**	Present	17/69 (24.6%)
	Absent	52/69 (75.4%)
***RET* somatic mutation**	Present	19/47 (40.4%)
	Absent	28/47 (59.6%)
***RAS* somatic mutation**	Present	4/42 (9.5%)
	Absent	38/42 (90.5%)
**Biochemical cure**	Yes	42/60 (70.0%)
	No	18/60 (30.0%)

### Tissue miR-375 Levels

Tissue miR-375 levels correlated weakly with CT levels at diagnosis (r^2^ = 0.095, P = 0.04), and tumor size (r^2^ = 0.14, P < 0.01).

Median tissue miR-375 levels were higher in males (0.044, IQR: 0.03–0.14 in males and 0.02, IQR: 0.008–0.06 in females, P = 0.04), in cases with positive lymph nodes (0.07, IQR: 0.03–0.17 in N1 patients; 0.02, IQR: 0.007–0.05 in N0 patients, P = 0.02), in patients with higher tumor stages at diagnosis (0.02, IQR: 0.007–0.05 for stages I+II; 0.08, IQR: 0.03–0.17 for stages III+IV; P = 0.02), and in patients not biochemically cured by the end of the follow-up (0.02, IQR: 0.008–0.03 in those who were biochemically cured; 0.06, IQR: 0.01–0.12 in those who were not; P = 0.03).

No correlations emerged with age at diagnosis (P = 0.42), or with somatic *RET* or *RAS* mutations (P = 0.43 and P = 0.71, respectively).

### Circulating miR-375 Levels

**Figure 1:**
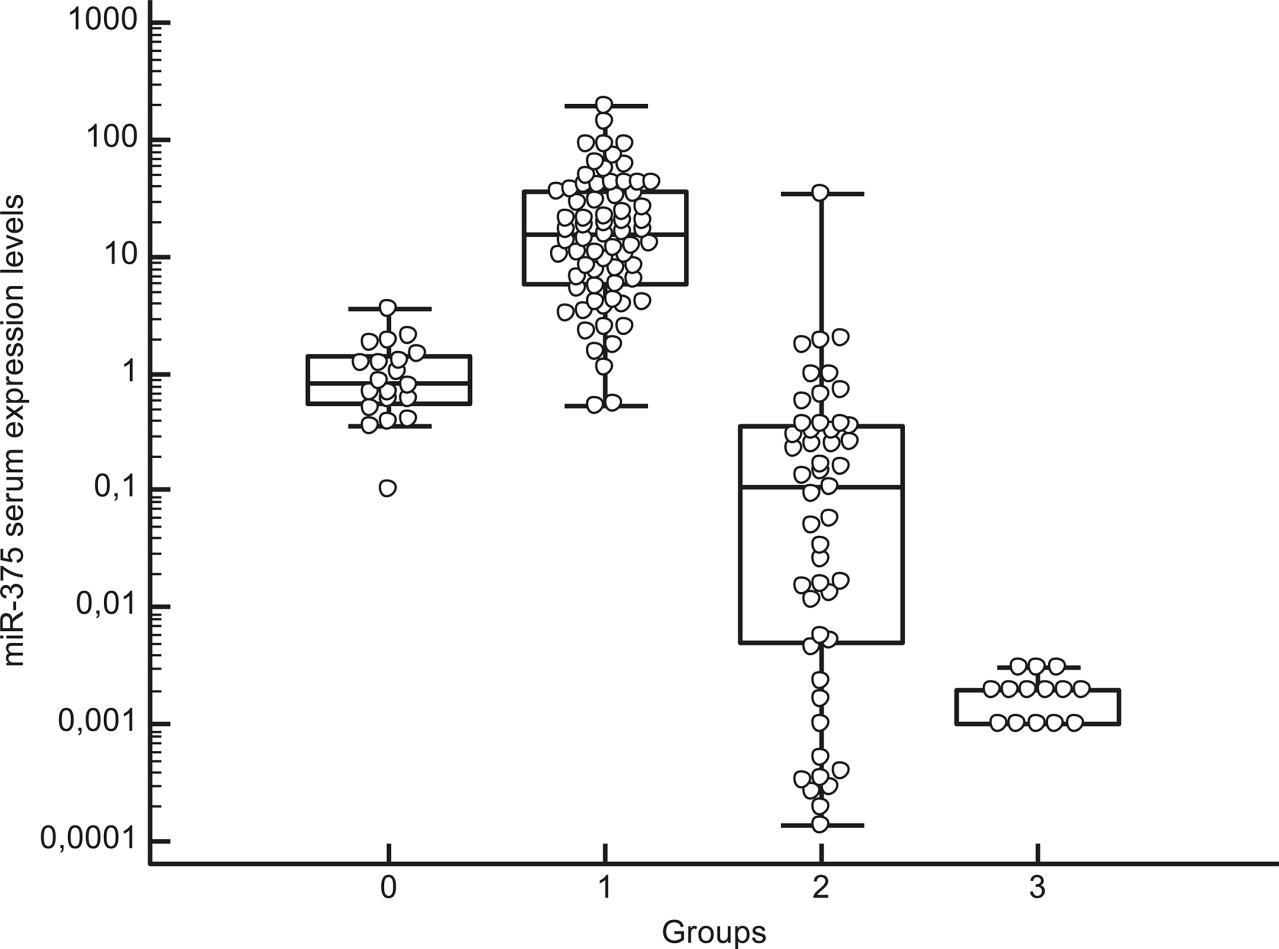
Box-whisker plot graph representing the medians, minimum and maximum values and interquartile ranges (IQR) of serum miR-375 levels in different groups: group 0: healthy subjects, group 1: MTC patients, group 2: patients with thyroid non-C-cell nodular diseases (non-CTN), group 3: pheochromocytoma patients. Values are represented on a logarithmic scale.

Median circulating miR-375 levels were higher in the serum of MTC patients (15.15, IQR: 5.82–36.51) than in the other patients and controls (P < 0.01), with no overlap in the IQR for these two groups’ miR-375 levels. The median miR-375 levels in the patients and controls were as follows: non-CTN patients 0.11, IQR: 0.005–0.36; pheochromocytoma patients: 0.002, IQR: 0.001–0.002; and healthy controls 0.84, IQR: 0.55–1.38. When the median values were compared, the MTC patients’ miR-375 levels were 101 times higher than in the non-MTC subjects (P < 0.01) (**Figure 1** and **Table 2**).

**Table 2:** *Post-hoc* test after Kruskal-Wallis analysis for median circulating miR-375 levels in the different groups: group 0: healthy subjects, group 1: MTC patients, group 2: patients with thyroid non-C-cell nodular diseases (non-CTN), group 3: pheochromocytoma patients.

Factor	n	Average Rank	Different (P < 0.05) from factor nr
**0 (healthy subjects)**	19	69.40	(1)(2)(3)
**1 (MTC)**	69	116.17	(0)(2)(3)
**2 (non-CTN)**	49	40.43	(0)(1)(3)
**3 (pheochromocytoma)**	14	17.36	(0)(1)(2)

Intriguingly, no correlation was found between serum and tissue miR-375 levels (P = 0.19).

Considering the MTC patients alone, there was no statistically significant difference in the median serum miR-375 levels by gender, although male patients had higher median levels than females (20.18, IQR: 5.21–40.23 *vs* 12.80, IQR of 6.93–23.26, P = 0.37). Nor was there any significant correlation between serum miR-375 levels and age at diagnosis (P = 0.58) or cancer size (P = 0.92), or between serum miR-375 levels and any presence of somatic *RET/RAS* mutations in MTC tissue (P = 0.35).

Median serum miR-375 levels were higher in patients with hMTC than in those with sMTC (23.77, IQR 15.98–40.81 *vs* 10.72, IQR: 4.18–27.49, P = 0.03), in N0 patients than in N1 patients (18.71, IQR: 9.79–35.12 *vs* 5.70, IQR: 2.48–12.17, P = 0.01), and in patients who were biochemically cured than in those who were not (19.56, IQR: 9.54–40.23 *vs* 4.67, IQR: 24.67–16.11, P = 0.02). There was no difference in the biochemical cure rate between hMTC and sMTC (P = 0.07), though a trend towards a worse prognosis emerged for sMTC: at the end of the follow-up, the biochemical cure rate was 28/44 (63.6%) cases of sMTC *versus* 14/16 (87.5%) cases of hMTC.

A trend towards higher median miR-375 levels was seen in patients with lower tumor stages at diagnosis (19.28, IQR: 9.79–37.4 for patients in stages I+II *vs* 7.45, IQR: 3.12–29.73 for those in stages III+IV), although the difference did not reach statistical significance (P = 0.05).

**Figure 2:**
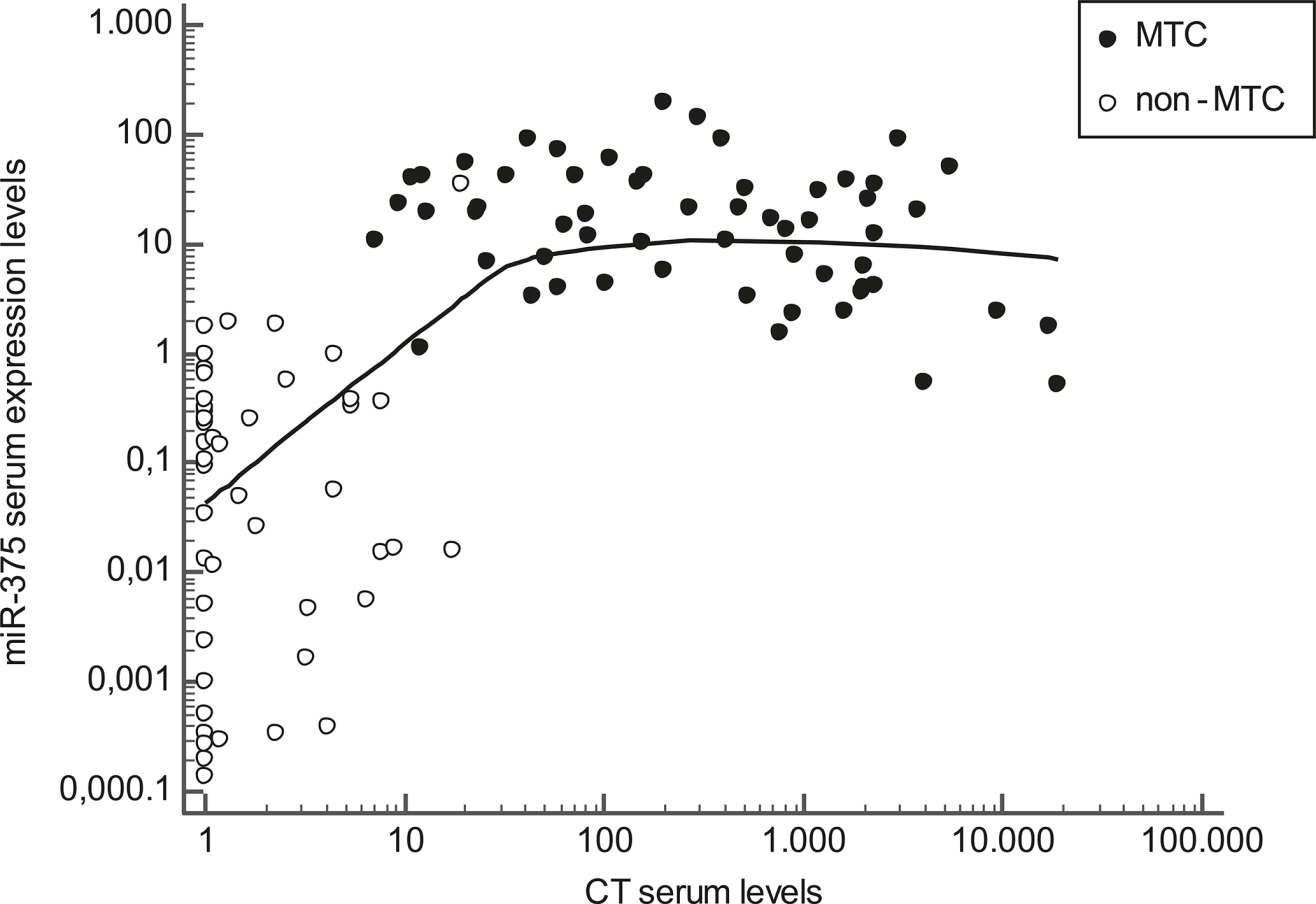
Scatter diagram representing the correlation between calcitonin (CT) serum levels (horizontal axis) and miR-375 serum levels (vertical axis) in MTC patients (black dots) and non-MTC patients (white dots). Values are represented on a logarithmic scale.

As for CT levels at diagnosis, median CT levels were higher in N1 than in N0 patients, (P< 0.01), in those with higher-stage tumors at diagnosis (stages III+IV) than in those with lower-stage disease (stages I+II) (P< 0.01), and in patients not biochemically cured at the end of the follow-up compared with those biochemically cured (P< 0.01). In the whole series of patients and controls, CT and serum miR-375 levels at diagnosis were correlated (r^2^ = +0.40, P< 0.01), but this correlation was linear only up to moderately high CT levels. Then circulating miR-375 levels tended to be lower the higher the CT levels. When only the MTC patients were considered, a weak negative correlation emerged between serum miR-375 levels and CT levels (r^2^ = −0.10, P = 0.01) (**Figure 2**).

**Figure 3:**
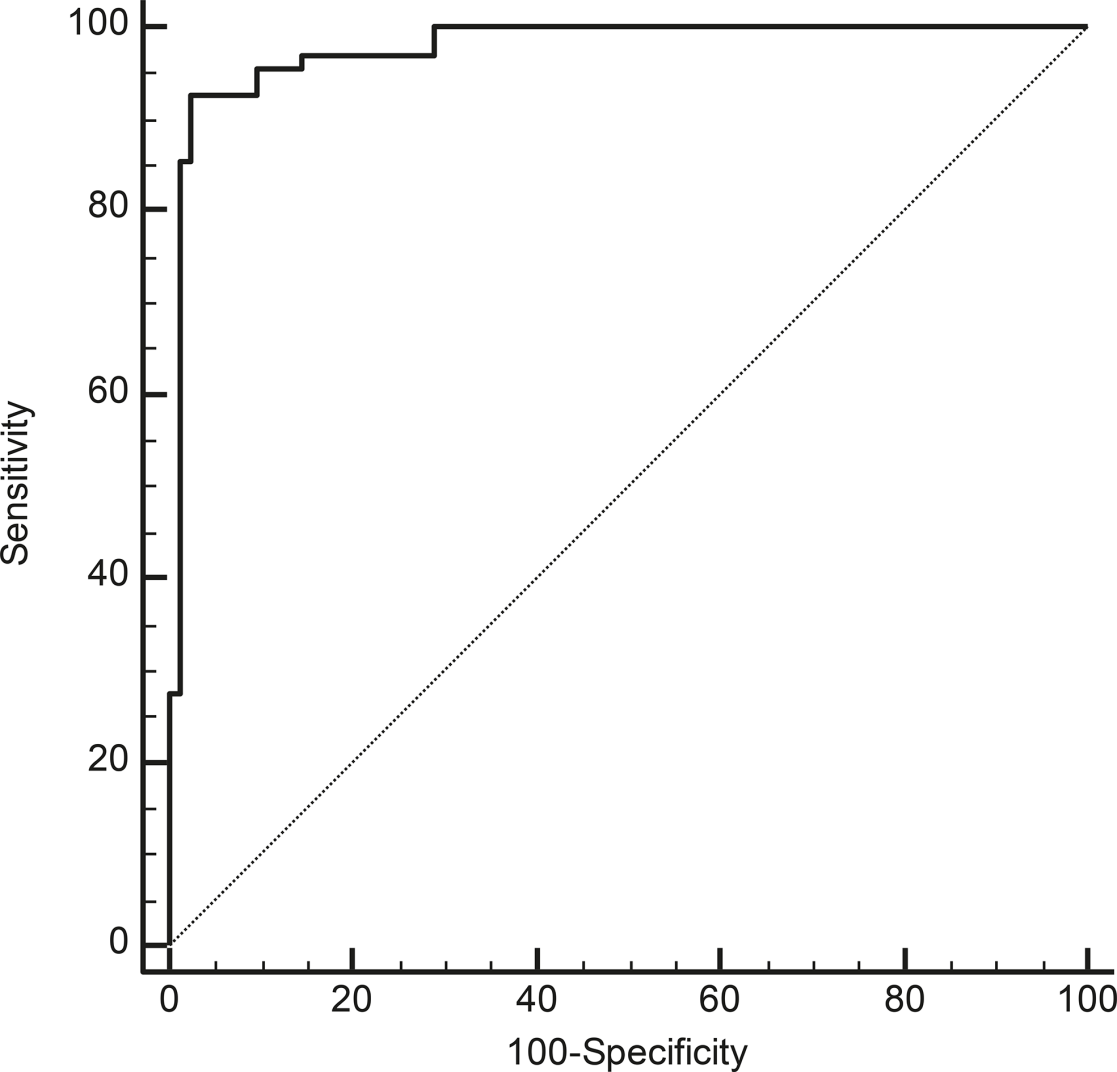
ROC curve analyses to identify the most accurate cut-off for miR-375 serum levels for differentiating between cases with and without MTC (P < 0.01; AUC: 0.978).

Receiver operating characteristic (ROC) curve analysis showed that a cut-off of >2.1 for serum miR-375 levels could distinguish between MTC patients and the other subjects with a sensitivity of 92.6%, a specificity of 97.6%, a positive predictive value (PPV) of 96.9%, and a negative predictive value (NPV) of 94.2% (AUC: 0.978, P < 0.01) (**Figure 3**).

Considering only the subjects whose CT levels at diagnosis were <100 ng/L (making a diagnosis of MTC more debatable), the cut-off for discriminating between cases with and without MTC was >0.97 (sensitivity 100%, specificity 93.6%, PPV 84.6%, NPV 100%, AUC 0.983, P< 0.01).

From our ROC curve analysis, it emerged that the best cut-off for serum miR-375 levels capable of identifying a patient with MTC unlikely to be biochemically cured at the end of the follow-up was ≤6.19. This cut-off was not very accurate, however (sensitivity 80.9%, specificity 55.6%, NPV 55.6%, PPV 81.0%, AUC 0.627, P = 0.185). MTC patients with serum miR-375 levels ≤6.19 therefore had a worse prognosis than those with higher levels: in the 60 evaluable MTC patients, 34/42 patients (80.9%) with serum miR-375 levels >6.19 were biochemically cured at the end of the follow-up, while this was true of only 8/18 patients (44.4%) with miR-375 levels below this cut-off (P< 0.01).

## Discussion

This study on a large series of subjects with various thyroid diseases demonstrated that circulating miR-375 can be useful in diagnosing MTC as there was no overlap between the levels measured in the groups with and without MTC. A previous study on a more limited series came to the same conclusion: Romeo *et al.* found higher miR-375 levels in the serum of 37 patients with persistent or recurrent metastatic MTC than in healthy controls (22). The same authors also found a greater *in situ* hybridization (ISH) reactivity for miR-375 on formalin-fixed, paraffin-embedded samples of MTC than on samples of CCH, while this reactivity was nil or very low on stromal and follicular thyroid cells. This could mean that miR-375 upregulation is a particular feature of C-cell biology. Having analyzed various benign or malignant thyroid tissues of follicular origin, our data certainly confirm serum miR-375 as a specific marker capable of identifying any cases of MTC. That said, a possible limitation of our study lies in that we were unable to analyze serum miR-375 levels for patients with CCH alone because they do not usually undergo thyroid surgery unless they carry *RET* germline mutations.

Our comprehensive analysis also included some patients with pheochromocytoma in an effort to see if circulating miR-375 could also serve as a marker in the diagnosis of neuroendocrine tumors other than MTC. Serum MiR-375 levels were almost undetectable in the patients with pheochromocytoma, however, thus confirming the specificity of this marker for C-cell neoplasia, at serum level at least. Intriguingly, serum miR-375 levels were found lower in patients with pheochromocytoma, also in comparison with healthy subjects. The reason at the basis of this result remains unknown. Unfortunately, our series did not include MEN2 patients with the contemporary presence of MTC and pheochromocytoma at the time of the withdrawal to understand how serum miR-375 could be found in this very peculiar setting. Further studies are needed to confirm our preliminary findings, testing circulating miR-375 levels in patients with other neuroendocrine tumors.

We identified a cut-off for circulating miR-375 of >2.1 that was able to discriminate between cases with and without MTC with a very good sensitivity and specificity. Serum CT is usually considered highly sensitive, but not very specific for the purposes of diagnosing MTC. In our opinion, miR-375 could be particularly useful in the case of moderately high CT levels and suspected thyroid nodular disease—a situation in which another confirmatory biochemical tool would be helpful for patients’ clinical management. In fact, a valid miR-375 cut-off for identifying MTC in subjects with moderately high CT levels (<100 ng/L) was also calculated (miR-375 >0.97).

In short, miR-375 performed well as a diagnostic tool in MTC patients, but further intriguing findings emerged on analyzing the correlations between the levels of miR-375 and CT. In our whole series of patients and controls, CT and miR-375 were directly related up to moderately high CT levels, but then (focusing on MTC sera) became inversely related (the higher the CT levels, the lower the miR-375 levels). Lower miR-375 levels in MTC sera were also associated with a worse prognosis. As mentioned earlier, miR-375 expression was also demonstrated on CCH samples. Pooling all these findings together in a view of neoplastic progression, we suggest that miR-375 upregulation occurs early in the process of C-cell neoplastic transformation, and may indicate an initially greater MTC differentiation that is subsequently lost as the cancer progresses and becomes more aggressive. In fact, high serum miR-375 levels were found in patients with no lymph node involvement and a low tumor stage at diagnosis, who were biochemically cured at the end of the follow-up. Intriguingly, these data contrast with findings at tissue level previously reported both by our group and elsewhere in the literature (23, 24). At tissue level, higher miR-375 levels were associated with more advanced clinicopathological features, and therefore with a tendency towards a more aggressive disease. In the light of this inconsistency vis-à-vis previous results, we also tested tissue miR-375 expression in a subgroup of paired MTC tissues in the present series, confirming that higher tissue miR-375 levels were associated with more advanced disease at diagnosis. As expected, higher CT levels in our series were also associated with higher tumor stages at diagnosis, lymph node involvement, and a lower biochemical cure rate, which goes to show that ours was not an “atypical” MTC series. The reason behind the mismatch between the prognostic significance of miR-375 in tissue and serum is still unclear. It was recently reported that exosomes (extracellular vesicles 30–100 nm in size) carry a non-random cargo of miRNAs, raising the hypothesis that miRNAs could also serve as intercellular paracrine and endocrine communicators. The delivery of miRNAs to surrounding and target tissues may therefore contribute to creating the milieu for cancer onset and dissemination (25). Exosome release is an active process that involves a number of finely regulated steps, from packaging to surface and adhesion molecules (26). We speculate that, as MTC becomes more aggressive and possibly dedifferentiated, its exosome delivery machine for miR-375 is lost, and levels of the latter in a patient’s serum consequently drop. Our findings regarding serum miR-375 levels differ from those obtained in the only other study that analyzed the prognostic value of miR-375 serum levels, in which Romeo *et al.* found higher circulating miR-375 levels in the sera of MTC patients with progressive disease, a greater tumor burden, and metastases. These discrepancies may be due to the marked differences between the two MTC series involved. Our series was tested prior to any surgery, and included cases of both high- and low-risk MTC, whereas Romeo *et al.* analyzed miR-375 on sera collected during the follow-up after surgery, and most of their cases involved persistent and recurrent progressive MTC (22).

To shed light on the possible role of miR-375 in the physiopathology of MTC, we investigated its association with *RET* and *RAS* mutations on a somatic and germinal level. No such associations emerged between somatic *RET* and *RAS* mutations and circulating miR-375 levels, consistently with our previous findings on tissue miR-375 expression levels (23), that we also replicated in the present series on MTC tissues. As regards *RET* germinal mutations, higher serum miR-375 levels were documented in hMTC than in sMTC. This finding should be taken with caution, however, as the data had a low level of statistical significance, whereas stronger data emerged on the association between higher miR-375 levels and higher biochemical cure rates. We surmise that the difference seen in miR-375 levels between hMTC and sMTC in our series could be due to a larger proportion of biochemically cured patients in the former group, rather than to any biological difference at a molecular level in the role of miR-375 role in these two categories of MTC patients.

In conclusion, our findings add to the previously published body of evidence suggesting that miR-375 can play a part in the complex landscape of MTC tumorigenesis. Further data are needed to better understand its role, its interaction with the known pathways involved in MTC, and its consequent diagnostic and prognostic value when measured in a given patient’s sera and tissues. Based on these intriguing and promising clinical results, further *in vitro* experiments will be necessary, and are now being conducted by our group. We are confident that a better understanding of the role of miRNAs in MTC could pave the way to the discovery of new molecular targets, especially in *RET*-negative MTC.

## Data Availability Statement

The original contributions presented in the study are included in the article/supplementary materials. Further inquiries can be directed to the corresponding author.

## Ethics Statement

The studies involving human participants were reviewed and approved by the ethical committee for clinical experimentation at Padua Hospital. The patients/participants provided their written informed consent to participate in this study.

## Author Contributions

SC and LB: study concept and design, analysis and interpretation, drafting of the manuscript, and final approval of the version to be published. CM and SB: study concept and design, supervision, final approval of the version to be published, and agreement with all aspects of the work. IP, AM, JM, CB, MI, and FG: substantial contributions to data acquisition and interpretation, critical revision of the manuscript, and final approval of the version to be published. MP and DF: substantial contributions to data acquisition. All authors: final approval of the version to be published, and agreement with all aspects of the work. All authors contributed to the article and approved the submitted version.

## Conflict of Interest

The authors declare that the research was conducted in the absence of any commercial or financial relationships that could be construed as a potential conflict of interest.
